# GO-PROMTO Illuminates Protein Membrane Topologies of Glycan Biosynthetic Enzymes in the Golgi Apparatus of Living Tissues

**DOI:** 10.1371/journal.pone.0031324

**Published:** 2012-02-21

**Authors:** Casper Søgaard, Anne Stenbæk, Sophie Bernard, Masood Hadi, Azeddine Driouich, Henrik Vibe Scheller, Yumiko Sakuragi

**Affiliations:** 1 Department of Plant Biology and Biotechnology, Faculty of Life Sciences, University of Copenhagen, Frederiksberg, Denmark; 2 Villum Kann Rasmussen Centre for ProActive Plants, Frederiksberg, Denmark; 3 Laboratoire de Glycobiologie et Matrice Extracellulaire-EA 4358, University of Rouen, Mont Saint Aignan, France; 4 Technologies Division, Joint BioEnergy Institute, Sandia National Laboratory, Emeryville, California, United States of America; 5 Feedstocks Division, Joint BioEnergy Institute, Lawrence Berkeley National Laboratory, Emeryville, California, United States of America; Institute of Molecular and Cell Biology, Singapore

## Abstract

The Golgi apparatus is the main site of glycan biosynthesis in eukaryotes. Better understanding of the membrane topology of the proteins and enzymes involved can impart new mechanistic insights into these processes. Publically available bioinformatic tools provide highly variable predictions of membrane topologies for given proteins. Therefore we devised a non-invasive experimental method by which the membrane topologies of Golgi-resident proteins can be determined in the Golgi apparatus in living tissues. A Golgi marker was used to construct a series of reporters based on the principle of bimolecular fluorescence complementation. The reporters and proteins of interest were recombinantly fused to split halves of yellow fluorescent protein (YFP) and transiently co-expressed with the reporters in the *Nicotiana benthamiana* leaf tissue. Output signals were binary, showing either the presence or absence of fluorescence with signal morphologies characteristic of the Golgi apparatus and endoplasmic reticulum (ER). The method allows prompt and robust determinations of membrane topologies of Golgi-resident proteins and is termed GO-PROMTO (for GOlgi PROtein Membrane TOpology). We applied GO-PROMTO to examine the topologies of proteins involved in the biosynthesis of plant cell wall polysaccharides including xyloglucan and arabinan. The results suggest the existence of novel biosynthetic mechanisms involving transports of intermediates across Golgi membranes.

## Introduction

The Golgi apparatus is an organelle that plays a central role in the assembly of glycans associated with various macromolecules (*i.e.* matrix polysaccharides, proteins, lipids) in eukaryotic cells [1], [2]. Biosynthesis of glycans requires concerted actions of enzymes and proteins including glycosyltransferases, modifying enzymes (*e.g.* methyltransferases, acetyltransferases, sulfatetransferases), nucleotide sugar transporters, and nucleotide sugar conversion enzymes, many of which are localized in the secretory pathway including endoplasmic reticulum (ER) and Golgi stacks. These enzymes and proteins must be oriented in the membrane so that the catalytic domains face the relevant sides of the membrane where the substrates are available and the products can be channeled to the enzymes and proteins in the proceeding steps during biosynthesis.

Because an experimental determination of protein membrane topology is often laborious, efforts have been directed towards bioinformatically predicting the topology of membrane proteins based on the structural and statistical evaluation of the amino acid sequences. The transmembrane domains of membrane proteins all have two common features: a hydrophobic middle section composed of mostly aliphatic amino acids [3], [4] and a flanking sequence composed of aromatic amino acids, mostly tryptophan and tyrosine [5]. With the inclusion of the positive-inside rules and machine-learning techniques, a dozen of algorithms for predicting topology has been established and is widely used, including poly-Phobius [6], HMMTOP [7], Prodiv-TMHMM [8], and TopPred [9].

These programs are said to correctly predict overall topologies of membrane proteins with an accuracy of ∼70% [10]–[14]. This means approximately one out of three or four membrane topologies predicted by a program is incorrect. Furthermore, different algorithms often lead to different topological predictions for the same protein. This is partly attributed to marginally hydrophobic regions, which are not predicted as transmembrane domains by many of the predictors due to low degrees of hydrophobicity but are inserted into the membrane due to long range tertiary interactions during protein folding [15].

A range of experimental methods for examining the topology of membrane proteins in the endomembrane system of eukaryotes has been developed, each with advantages and drawbacks. Biochemical approaches include recognition of glycosylation mapping [16], cysteine substitutions [17], and proteinase susceptibility assays [18]. These methods are invasive and require cell disruption, thus resulting in a loss of information about the subcellular localizations of the tested proteins.

Several non-invasive methods have been reported based on the use of green fluorescent protein (GFP) and its variants for detection of the topology of proteins localized in the endomembrane system in living cells. A classical biochemical protease susceptibility assay [19], [20] coupled with fluorescent protein fusions was used to determine protein membrane topology in a variety of organelles including the Golgi apparatus, mitochondria, peroxisome, and autophagosomes [21]. The method has been shown to work robustly in human cell cultures; however, its applicability to intact, whole tissue samples of different biological systems has not been addressed. Bimolecular fluorescent complementation (BiFC) was used to determine the membrane orientation of N- and C-termini of a protein localized to the ER membranes in living plant tissues [22]. This method relies on cytosolic and ER lumenal reporters that detect the tagged amino acid termini if localized to the cytosol or ER lumen, respectively. pH-Dependent fluorescent intensity of yellow fluorescent protein (YFP) was used to determine the membrane topology of plasma membrane-associated proteins in plant tissues [23]. Due to the low pH values (ca. 5–6) in the apoplast, YFP fluorescence is decreased when exposed to the apoplast while a robust signal is detectable when exposed to the cytosol. The absence of signal is inferred, but not directly shown, as apoplastic localization. A redox-sensitive GFP (roGFP) has been utilized to probe the ER-localized membrane protein topology by exploiting the glutathione redox gradient across the ER membrane [24]. This method provides ratiometric outputs that distinguish the cytosolic and the ER luminal localizations of the fluorescent tags, but it requires an advanced filter setup and post-imaging processing. The application of any of these methods to Golgi lumen has not been demonstrated.

The lack of a reliable method allowing topology determinations of Golgi membrane proteins in complex living tissues prompted us to establish a method based on the BiFC system. GO-PROMTO (GOlgi PROtein Membrane TOpology analysis), produces binary signals illuminating the orientation of the N- and C-termini of the tested proteins in the Golgi apparatus. We have applied the method to determine the membrane topologies of eleven enzymes involved in the biosynthesis of cell wall polysaccharides in the higher plant Arabidopsis. The data reveal new insights into the mechanisms controlling the assembly of complex glycans within the Golgi membranes.

## Results

### Establishment of the GO-PROMTO method

**Figure 1:**
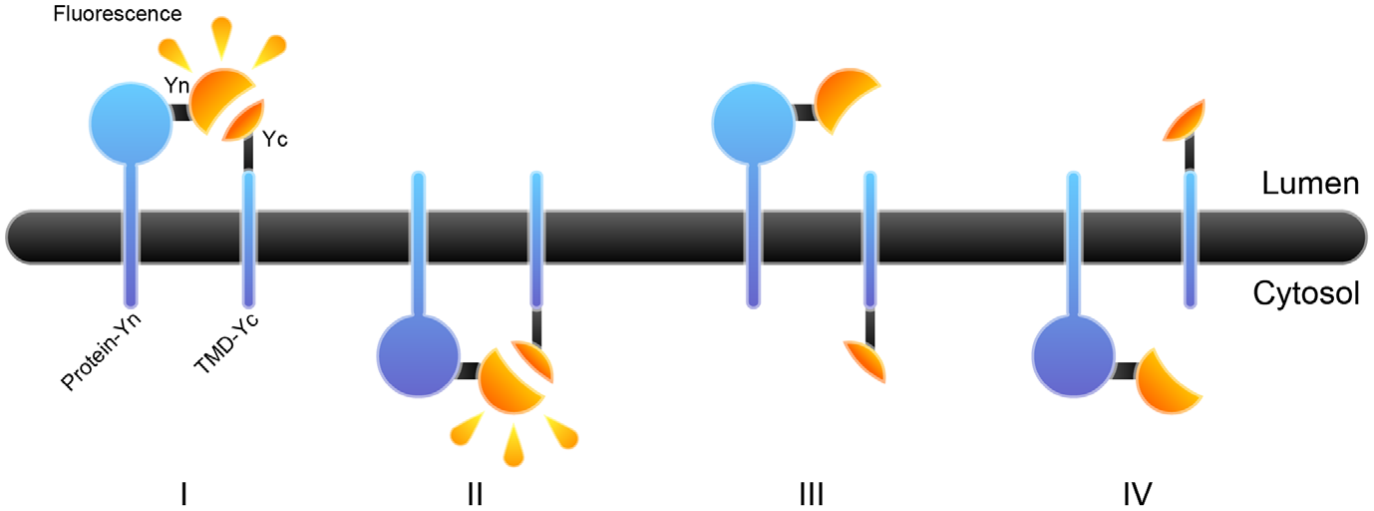
Illustrations of the principle of GO-PROMTO. Fluorescent complementation occurs when the two complementary domains of split YFP (Yn and Yc) fused to the test protein and TMD are present on the same side of the membrane (I and II), whereas no fluorescence occurs if they are on the opposite sides of the membrane (III and IV).

There are four main criteria that need to be met for a topology reporter to prove robust: i) the signal-to-noise ratio must be high; ii) the topology reporter must recognize the luminal localization of protein termini of tested proteins; iii) the topology reporter must be promiscuous and recognize every tested protein; iv) the method must be simple, preferably inexpensive, and easily adapted to standard laboratories. In order to establish a method that produces excellent signal-to-noise ratio in the Golgi apparatus, we used BiFC. YFP (Venus) was split in two parts, “Yn” (amino acids 1–155 of YFP) and “Yc” (amino acids 156–238 of YFP) as previously reported [25]. The individual halves are not fluorescent, while the two parts can reconstitute if they are in the same microcompartment and emit fluorescence with a maximum peak at 535 nm upon excitation at 514 nm. Therefore the system gives either “on” or “off” output (Figure 1).

A series of topology reporters were generated by using a truncated rat sialyltransferase sequence. Sialyltransferase has the canonical type II membrane topology with N- and C-termini presented in the cytosol and the Golgi lumen, respectively. A truncated sialyltransferase consisting of the N-terminal 52 residues containing a transmembrane domain is widely used as a Golgi marker (hereafter “TMD”) [26]–[28]. In-frame fusions of the TMD and the split YFP halves were made in order to create the GO-PROMTO topology reporters. Two with the sensor domain facing the cytosol (“cytosolic-TMD reporters”; Yn/Yc-TMD) and two lumen-oriented topology reporters with the sensor domains localized in the Golgi lumen (“lumenal-TMD reporters”; TMD-Yn/Yc).

**Figure 2:**
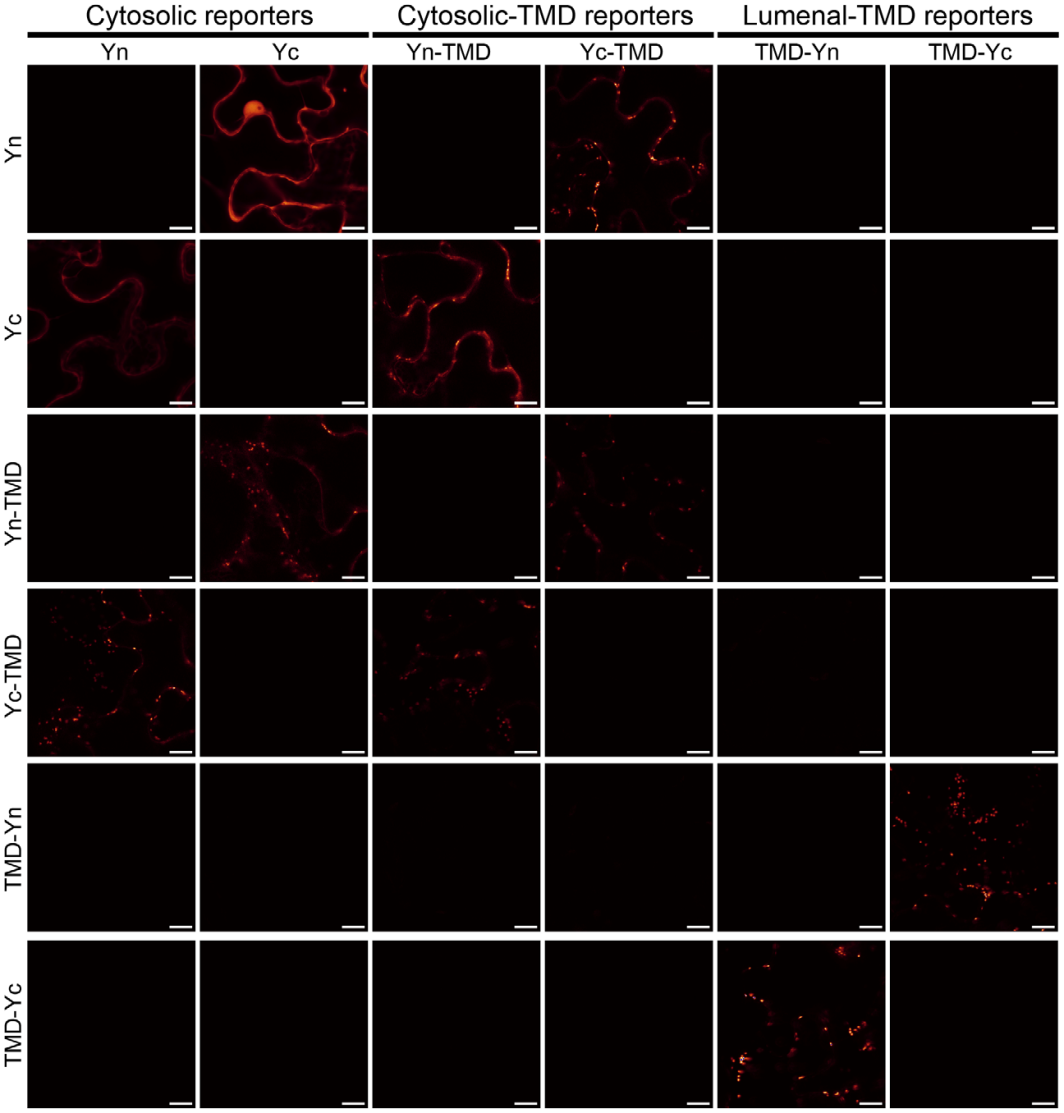
Fluorescence complementation among the GO-PROMTO reporters expressed in the whole leaf tissue of *Nicotiana benthamiana* at 3 days DPI. The GO-PROMTO reporters were co-expressed and fluorescence complementation was examined by CLSM upon excitation at 514 nm and detection between 529 nm and 599 nm. All scale bars indicate 10 µm. At least two individual experiments were performed for each combination with the similar results. Raw images are shown.

When introduced individually into leaves of the tobacco plant *Nicotiana benthamiana* via transient transfection [29], none of these reporters alone produced detectable signals as shown in Figure 2. When expressed in combinations, the lumenal-TMD reporters complemented fluorescence signals characteristic of the Golgi apparatus as revealed by CLSM. Similarly, the cytosolic-TMD reporters complemented fluorescence and showed both the Golgi apparatus and ER-like signals. No complementation was observed when the lumenal- and cytosolic-TMD reporters were co-expressed. Yn and Yc without fusion proteins were included as reporters that localize in the cytosol without a membrane anchor (“cytosolic reporters”). The cytosolic reporters complemented fluorescence with the cytosolic-TMD reporters but not with the lumenal-TMD reporters under the current conditions (Figure 2). In rare occasions, we have noticed that negative interactions gave weak and sporadic positive signals if reporters were hyperexpressed and if the gain value of the microscope was set high. This could be due to a mistargeting, misinsertion, and/or flipping of these reporters across the membrane. It is noteworthy that ER signals were detectable even though the topology reporters were based on the well-defined Golgi marker, which is likely due to high-level expression of the reporters. These observations suggest that the topology reporters can be used to detect topologies of both Golgi- and ER-localized proteins.

**Figure 3:**
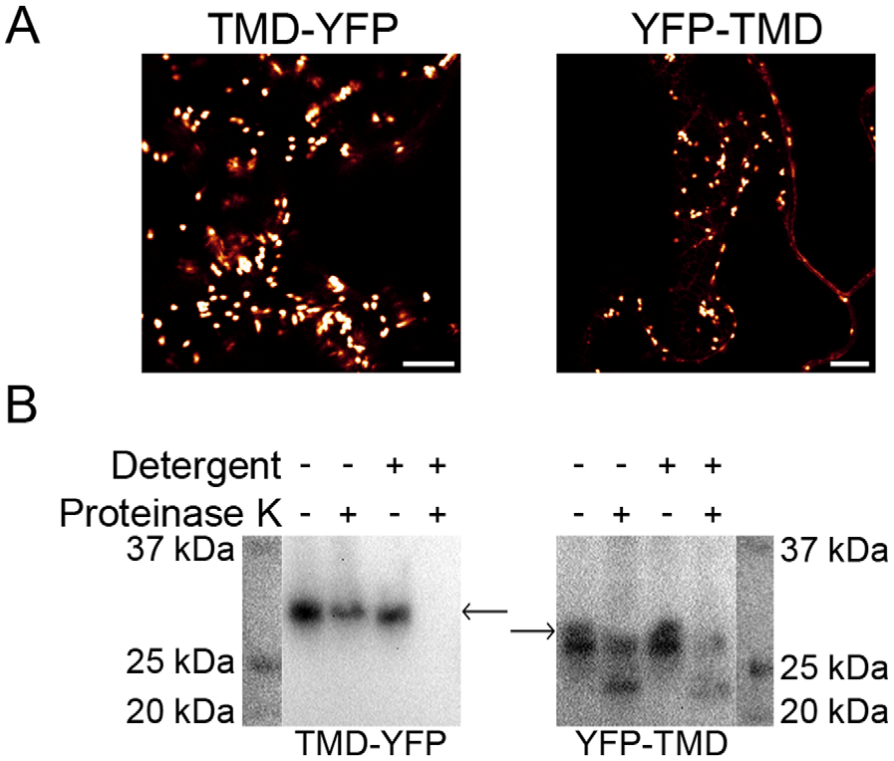
Proteinase protection assay of the TMD fused to YFP at the N- and C-terminus. **A**. TMD-YFP and YFP-TMD were transiently expressed in *Nicotiana benthamiana* whole leaves and CLSM was carried out at 3 DPI. Both fusion proteins show predominantly Golgi apparatus localization. Scale bars, 10 µm. **B**. Immunoblot analysis of the TMD-YFP and YFP-TMD after treatment with proteinase K in the presence or absence of TritonX-100. Molecular masses of TMD-YFP and YFP-TMD are estimated to be 33.8 kDa and 34.5 kDa, respectively (Compute pI/Mw server at Expasy.org). The full-length fusion proteins are indicated with the arrows. TMD-YFP is degraded only in the presence of detergent and protease indicating Golgi lumenal orientation of YFP tag. YFP-TMD was degraded regardless of detergent addition, indicating cytosolic orientation of YFP tag. Partial degradation of the YFP was observed for YFP-TMD (bands immediately below the full-length YFP). At least two individual experiments were performed for each combination with the similar results. Raw images are shown.

Proteinase susceptibility assays were performed in order to validate the membrane orientation of the TMD fusion proteins. TMD fused to the full-length YFP at N- and C-termini, YFP-TMD and TMD-YFP, respectively, were transiently and individually expressed in *N. benthamiana* leaves (Figure 3A). Microsomes isolated from these leaves were treated with proteinase K in the absence or presence of the detergent Triton X-100. Degradation of YFP was monitored by SDS-PAGE and immunoblotting with anti-GFP antibody which also recognizes YFP. The YFP tag in TMD-YFP was degraded by the protease only when the detergent was present, while the YFP tag in YFP-TMD was degraded regardless of the presence or absence of the detergent (Figure 3B). Untreated YFP-TMD migrated slightly faster than TMD-YFP in SDS-PAGE and an additional product with slightly lower apparent molecular mass that was resistant to proteinase K was observed. These results may indicate that the YFP tag, when localized to the cytosolic side of the membrane, is likely to be partially degraded. The combined results unequivocally demonstrate that the YFP tag localizes to the cytosol when fused to the N-terminus of TMD, whereas it localizes to the Golgi lumen when fused to the C-terminus of TMD. These results demonstrate that the reporters detect the cytosolic and lumenal orientations of the protein termini in the Golgi apparatus and ER with binary signal outputs.

### Validating the robust performance of GO-PROMTO

In order to evaluate the performance of GO-PROMTO, the membrane topologies of Golgi-localized, predicted type II membrane proteins involved in cell wall polysaccharide biosynthesis in the model plant *Arabidopsis thaliana* were studied. RGXT2 is a 1,3-α-D-xylosyltransferase involved in rhamnogalacturonan II biosynthesis, whereas UXS2 is a UDP-glucuronic acid decarboxylase responsible for xylose biosynthesis. Both enzymes have been shown to localize to the Golgi apparatus by using C-terminal GFP fusions coupled to live-cell imaging [30]–[32]. IRX10-like (IRX10L) is a member of the GT47 glycosyltransferase family involved in xylan biosynthesis [33], [34]. IRX10L has been suggested to localize to a Golgi fraction in a proteomics study [35].

**Figure 4:**
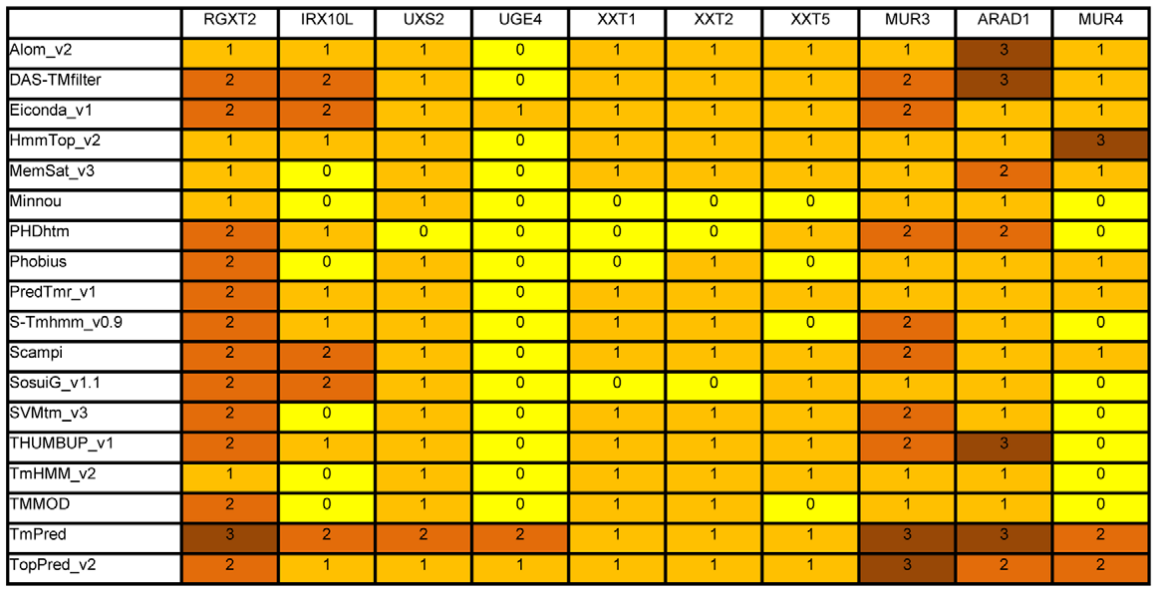
Prediction of the number of transmembrane domains for selected cell wall biosynthetic enzymes. The data compiled in Aramemnon (http://aramemnon.uni-koeln.de/) is summarized. Numbers indicate the number of transmembrane domains.

**Figure 5:**
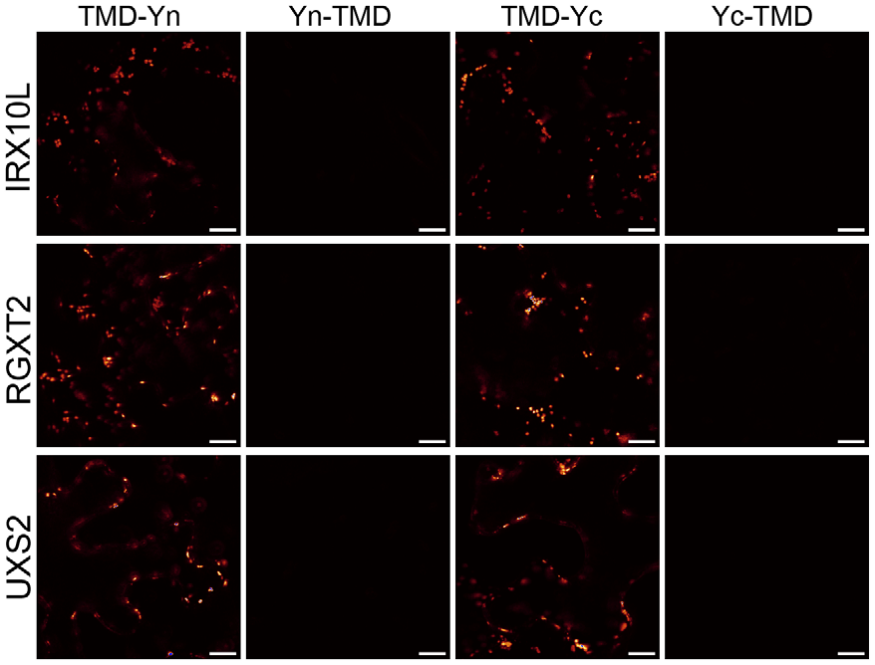
GO-PROMTO analysis of membrane proteins (IRX10L, RGXT2, and UXS2) known to be involved in cell wall polysaccharide biosynthesis in plant. The C-terminal Yc fusion of each of the proteins was co-expressed with TMD-Yn (1^st^ column) or Yn-TMD (2^nd^ column) and the C-terminal Yn fusion of each of the protein was co-expressed with TMD-Yc (3rd column) or Yc-TMD (4^th^ column). The fluorescence complementation with the lumenal reporters and the lack of same with the cytosolic reporters indicate Golgi lumenal orientation of the IRX10L, RGXT2 and UXS2 catalytic domains. Scale bars, 10 µm. At least two individual experiments were performed for each combination with the similar results. Raw images are shown.

The number of predicted transmembrane domains in these proteins varied considerably, between zero and three, depending on the prediction program (Figure 4). For example, TmHMM (ver. 2) predicts that RGXT2 and UXS2 contain single transmembrane domains while IRX10L contains no transmembrane domain. In contrast, TopPred (ver. 2) predicts that RGXT2 contains two transmembrane domains and IRX10L and UXS2 contain single transmembrane domains. Assuming that the N-terminus is placed in the cytosol, the presence of a single transmembrane domain would place the C-terminal catalytic domain in the Golgi lumen, whereas the absence (zero transmembrane domain) or the presence of two transmembrane domains would place it in the cytosol or at the cytosolic surface of the Golgi membrane, respectively. The cytosolic- and lumenal-TMD reporters were co-expressed with these proteins, fused with Yn and Yc at their C-termini, in order to address the membrane topology of these proteins. In all cases, fluorescence complementation was observed between the test proteins and the lumenal-TMD reporters but not between the test protein and the cytosolic-TMD reporters (Figure 5). Furthermore, the fluorescence signals detected were typical of Golgi localization. These results clearly demonstrate that the C-termini of these proteins are localized to the Golgi lumen.

**Figure 6:**
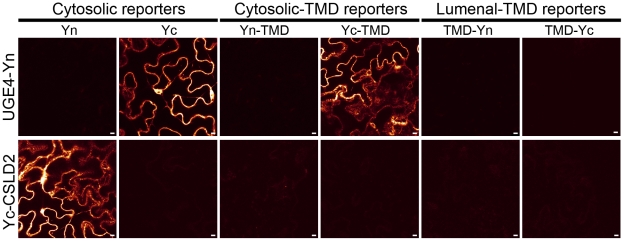
GO-PROMTO analysis of cytosolic (UGE4) and multimembrane spanning protein (CSLD2) known to be involved in cell wall polysaccharide biosynthesis in plant. UGE4 was fused to Yn at the C-terminus and CLSD2 was fused to Yc at the N-terminus and were transiently expressed in *Nicotiana benthamiana*. Observation was carried out by CLSM at 3 DPI. Scale bars, 10 µm. At least two individual experiments were performed for each combination with the similar results. Raw images are shown.

Next, a cytosolic protein and a multimembrane-spanning protein were tested against all six topology reporters. UGE4 is a UDP-glucose 4-epimerase and has previously been shown to be located in the cytosol [36]. As expected, UGE4-Yn did not complement fluorescence with the lumenal TMD reporters whereas it complemented fluorescence with the cytosolic TMD and soluble reporters (Figure 6). The similar results were obtained for the N-terminally tagged UGE4 (Yc-UGE4) (data not shown). Cellulose Synthase Like D2 (CSLD2) is a multimembrane-spanning protein that localizes to the Golgi apparatus and has been postulated to be involved in mannan synthesis [37]. The N-terminus of CSLD2 has been shown to be located in the cytosol [38]. Yc-CSLD2 complemented fluorescence with the cytosolic reporters whereas it did not complement fluorescence with the lumenal-TMD reporters (Figure 6). When Yn-TMD, a cytosolic-TMD reporter, was used, the detected signal was very weak, which is likely to be due to partial degradation of the N-terminus of Yn (Figure 3). It appears that the degree of degradation of the Yn-TMD reporter is highly variable. Taken together, the results presented in Figure 5 and 6 demonstrate that the five out of six topology reporters (Yn, Yc, Yc-TMD, TMD-Yn, TMD-Yc) specifically and robustly detect the topologies of the tested protein termini.

### Membrane topologies of xyloglucan-biosynthesizing enzymes

GO-PROMTO was used to determine the membrane topology of enzymes involved in the biosynthesis of xyloglucan. Xyloglucan is a major hemicellulosic polysaccharide of primary cell wall, synthesis of which occurs in the Golgi apparatus [39]–[41]. In Arabidopsis, CSLC4 is thought to catalyze the synthesis of β-1,4-linked glucan backbone [42]. This glucan backbone is decorated with the side-chain α-1,6-xylosyl residues by XXT1, XXT2 and XXT5 [43], [44], and can be further substituted with β-1,2-galactosyl and α-1,2-fucosyl residues by MUR3 and FUT1, respectively [43], [45]–[47]. All these enzymes have been shown to localize to the Golgi cisternae [48], [49]. A previous study has shown that the N- and C-termini and the catalytic domain of CSLC4 localize to the cytosolic side of the Golgi membrane [48]. This indicates that the backbone synthesis of xyloglucan occurs at the cytosolic face of the Golgi membrane and raises a question about the subcellular compartment in which the side-chain modifications occur.

**Figure 7:**
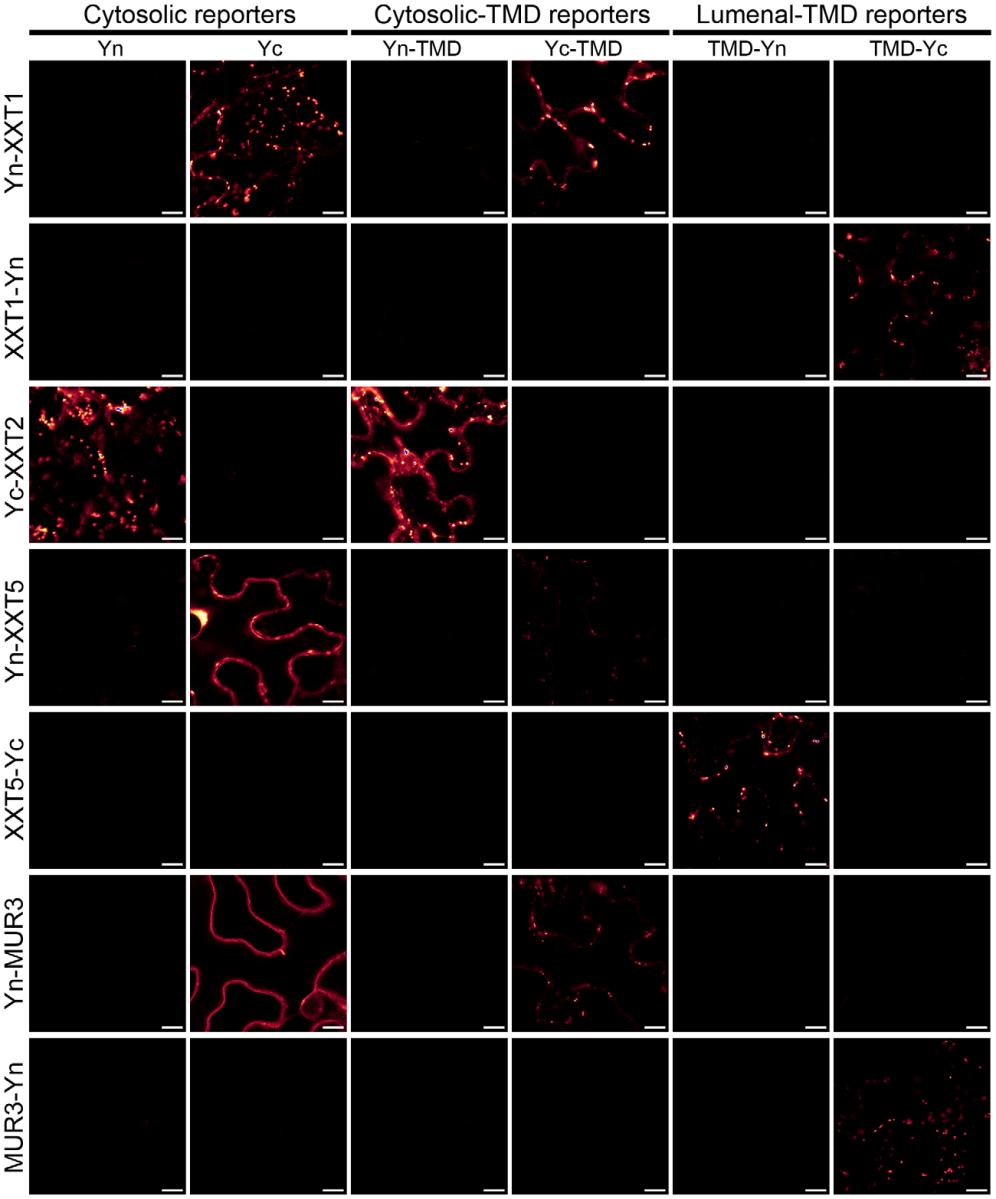
GO-PROMTO analysis of enzymes involved in the xyloglucan biosynthetic pathway. All proteins were fused to GO-PROMTO tags in the N-terminus. Additionally XXT1, XXT5 and MUR3 were fused to Yn or Yc at the C-termini. In all cases, the N-terminal of the proteins was found to localize to the cytosol by only reacting with the cytosolic GO-PROMTO reporters, concurrent with bioinformatic predictions. Furthermore, C-terminal fusions of MUR3, XXT5 and XXT1 showed Golgi lumenal localization. These fusion proteins were transiently expressed in *Nicotiana benthamiana*. Observation was carried out by CLSM at 3 DPI. Scale bars, 10 µm. At least two individual experiments were performed for each combination with the similar results. All of the images were processed identically by using Adobe Photoshop CS3.0 Extended v10.0.

The membrane topologies of the side chain biosynthetic enzymes have only partially been elucidated: it has been shown that the terminal fucosylation by FUT1 occurs in the Golgi lumen [50] and that XXT1 has its catalytic domain in the Golgi lumen [38]. Therefore we have investigated the membrane topology of four side-chain biosynthetic enzymes, XXT1, XXT2, XXT5 and MUR3. XXT1, XXT2 and XXT5 are predicted to contain zero or one transmembrane domain, while MUR3 is predicted to contain one, two and three transmembrane domains (Figure 4). Again, assuming cytosol-localized N-termini, odd-numbered transmembrane domains would place the C-terminal catalytic domains of these proteins in the Golgi lumen whereas zero and even-numbered transmembrane domains would place the catalytic domains in the cytosol. GO-PROMTO analysis was carried out in order to gain insight into the membrane topology of these side-chain synthesizing enzymes. N-terminal fusions of XXT1, XXT2, XXT5 and MUR3 complemented fluorescence with the cytosolic and cytosolic-TMD reporters, whereas they did not complement fluorescence with the lumenal-TMD reporters (Figure 7). This suggests that their N-termini are localized to the cytosol. The C-terminal fusions of XXT1, XXT5 and MUR3 did not complement fluorescence with the cytosolic and cytosolic-TMD reporters whereas they complemented fluorescence with the lumenal-TMD reporters and gave rise to signals characteristics of the Golgi apparatus (Figure 7). These results indicate that the catalytic domains of XXT1, XXT5 and MUR3 are localized to the Golgi lumen.

### Membrane topologies of arabinan-biosynthesizing enzymes

Arabinan is mostly associated with the side chain of rhamnogalacturonan I. It consists of α-1,5-linked linear oligoarabinosaccharide with α-1,3- or α-1,2-linked branches also consisting of arabinose. Four gene products, MUR4, RGP1, RGP2 and ARAD1 have previously been shown to be involved in arabinan biosynthesis. MUR4 catalyzes the conversion of UDP-xylose to UDP-arabinopyranose [51]. RGP1 and RGP2 convert UDP-arabinopyranose to UDP-arabinofuranose [52], [53]. ARAD1, a putative arabinosyltransferase, is involved in the incorporation of UDP-arabinofuranose into the growing arabinan structure [54].

It has been shown that RGP1 and RGP2 are located in the cytosolic face of the Golgi apparatus [52], [53] whereas ARAD1 and MUR4 are thought to be membrane anchored and have been shown to localize to the Golgi membrane [29], [51]. A previous study has indicated that MUR4 presents a predicted type II membrane topology [51]. However, a closer inspection of topology predictions revealed that the number of transmembrane domains vary considerably from no transmembrane domain (Minnou, PHDhtm, S-Tmhmm_v0.9, TmHMM_v2, SosuiG_v1.1, SVMtm_v3, THUMBUP_v1, TMMOD) to one transmembrane domain (MemSat_v3, Phobius, PredTmr_v1, Scampi, Alom_v2, DAS-TMfilter, Eiconda_v1), two transmembrane domains (TmPred, TopPred_v2), and three transmembrane domains (HmmTop_v2) (Figure 4). Furthermore, the membrane topology predictions of ARAD1 also vary from one transmembrane domain (TmHMM_v2, TMMOD, SVMtm_v3, SosuiG_v1.1, Scampi, S_Tmhmm_v0.9, PredTmr_v1, Phobius, Minnou, HmmTop_v2, Eiconda_v1), two transmembrane domains (PHDhtm, TopPred_v2, MemSat_v3), and to three transmembrane domains (Alom_v2, DAS-TMfilter, TMUMBUP_v1, TmPred) (Figure 4).

**Figure 8:**
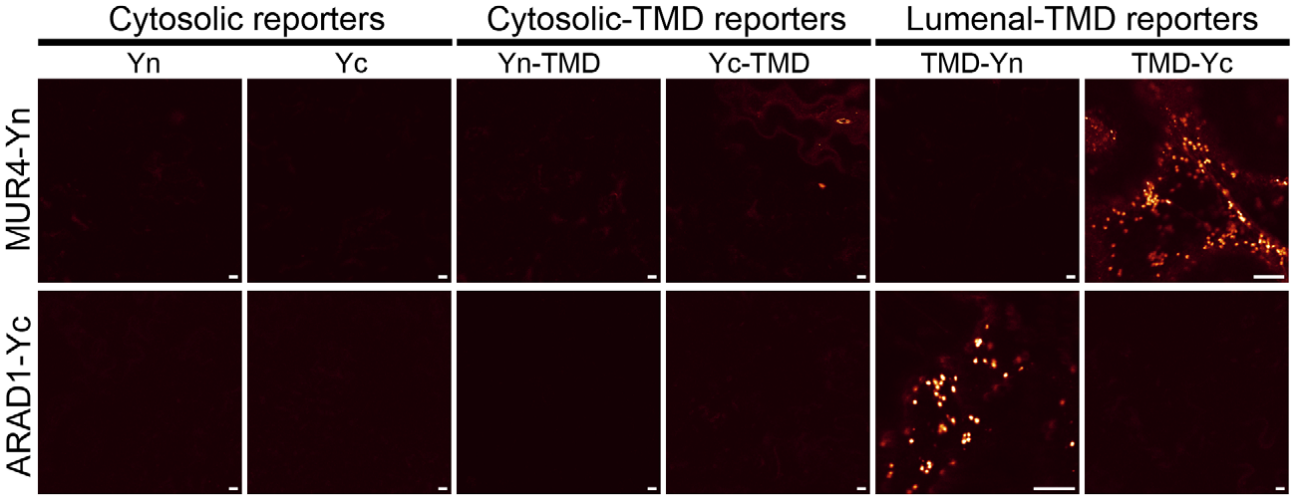
GO-PROMTO analysis of ARAD1 and MUR4, putative arabinosyltransferase and UDP-xylose/arabinose epimerase. ARAD1 and MUR4 fused with the Yc and Yn tags, respectively, at the C-termini were transiently expressed in *Nicotiana benthamiana*. Fluorescence complementation with lumenal reporters suggests Golgi-lumenal localization of ARAD1 and MUR4 C-termini. Observation was carried out by CLSM at 3 DPI. Scale bars, 10 µm. At least two individual experiments were performed for each combination with the similar results. Raw images are shown.

GO-PROMTO analysis was carried out for ARAD1 and MUR4 (Figure 8). The cytosolic and cytosolic-TMD reporters did not complement fluorescence with the C-terminal fusions of ARAD1 and MUR4. In contrast, the lumenal-TMD reporters complemented fluorescence with both the C-terminal fusions of ARAD1 and MUR4. These results clearly demonstrate that the C-terminal catalytic domain of ARAD1 and MUR4 are located in the Golgi lumen.

## Discussion

We have developed GO-PROMTO, a protein membrane topology method for fast and easy determination of the membrane topologies of proteins in the Golgi apparatus in living tissues. A series of topology reporters were generated and validated by live cell imaging and by the protease protection assay. The method is non-invasive, gives robust binary signal output, does not require further chemical treatments, and detects both the cytosolic and lumenal localization of the tagged termini for Golgi-resident proteins. GO-PROMTO was used to determine the membrane topologies of eleven proteins involved in plant cell wall biosynthesis as detailed below.

The membrane topology of the cell wall polysaccharide biosynthesis in plants is still not well understood. The previous topological studies of glucan synthases that catalyze the formation of the backbone of xyloglucan presented contradictory conclusions. In pea it was suggested that the catalytic site of a glucan synthase I, thought to synthesize the backbone glucan chain in the xyloglucan biosynthesis, is placed in the Golgi lumen [55]. In contrast a later study based on heterologous expression in *Pichia pastoris* provided compelling evidence that the catalytic site of the Arabidopsis glucan synthase CSLC4 is placed in the cytosol [48]. In this case, the synthesized glucan chain must be transported to the Golgi lumen where xyloglucan epitope is found [39]. The subcellular site(s) of side-chain synthesis have not yet been addressed except for that the terminal fucosylation has been shown to occur in the Golgi lumen [50] and that the putative catalytic domain of XXT1 has been shown to localize to the Golgi apparatus by protease protection assay [38]. By using GO-PROMTO we have validated the method by showing the C-terminus of XXT1 is located in the Golgi lumen, as previously shown [38] and have determined the membrane topology of enzymes responsible for two of the remaining steps of the side-chain synthesis (XXT5 and MUR3). Our results demonstrated that XXT5 and MUR3 have their C-terminal catalytic domains in the lumen (Figure 7). These results, together with the previous studies, indicate that the glucan backbone, if synthesized in the cytosol, is translocated across the membrane and that the side-chain modifications including the xylosylation by XXT1 and XXT5, galactosylation by MUR3 and the terminal fucosylation occur in the Golgi lumen.

The recent discovery that an enzymatic step in the pectic arabinan biosynthesis occurs in the cytosol has lead to an intriguing hypothesis about the mechanism of pectic arabinan biosynthesis. Scheller and colleagues have identified that the RGP1 and RGP2 encode UDP-arabinose mutases that are essential for the generation of UDP-arabinofuranose, an intermediate in arabinan biosynthesis [53]. Interestingly the authors also identified that RGP1 and RGP2 localize to the cytosolic surface of the Golgi apparatus as well as in the cytosol. On the other hand, it is generally regarded, though without experimental evidence, that the MUR4 and ARAD1 enzymes, involved in the arabinan biosynthesis at the preceding and proceeding steps of RGP1 and RGP2, respectively, posses the type II membrane topology with the catalytic C-termini located in the Golgi lumen. This apparent topology conundrum of arabinan biosynthesis prompted us to examine the subcellular localization of the C-termini of MUR4 and ARAD1.

**Figure 9:**
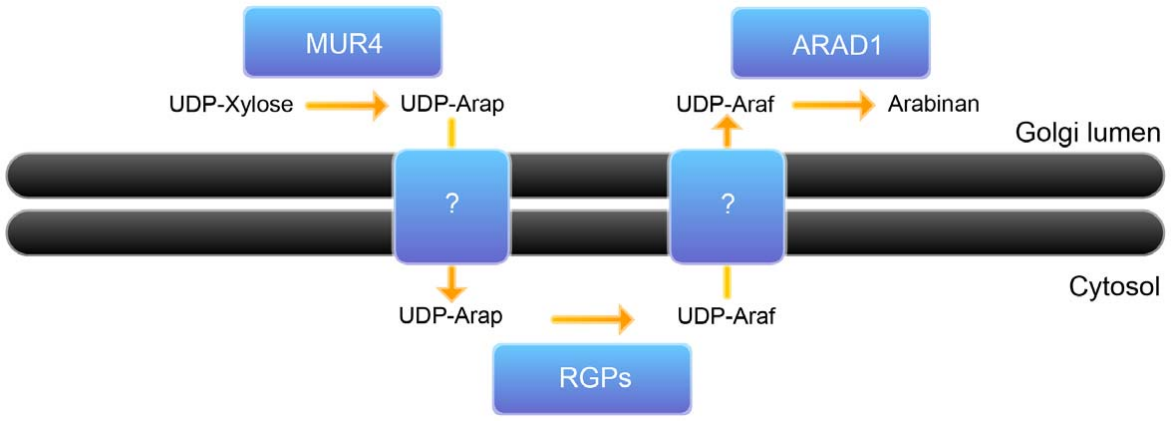
A topology model of Arabinan biosynthesis. UDP-arabinopyranose (UDP-Ara*p*) generated by MUR4 in the Golgi lumen is transported to the cytosol possibly by a transporter; UDP-Ara*p* is converted to UDP-Ara*f* by RGP1 and RGP2 in the cytosol; UDP-Ara*f* is transported from the cytosol to the Golgi lumen possibly by a transporter; UDP-Ara*f* is incorporated into arabinan by glycosyltransferase(s) likely including ARAD1.

Topology predictions of MUR4 and ARAD1 were found to be highly variable. Based on the topology prediction, three scenarios were considered: i) the catalytic domains of MUR4 and ARAD1 localize to the cytosol; ii) the catalytic domain of MUR4 localizes to the cytosol whereas that of ARAD1 localizes to the Golgi lumen; iii) the catalytic domains of both MUR4 and ARAD1 localize to the Golgi lumen. To test these hypotheses, we have carried out GO-PROMTO analysis of MUR4 and ARAD1. Our results clearly showed that the C-termini of both proteins are located in the Golgi lumen, therefore the third scenario is likely to be the case. This mode of biosynthesis, requiring a shuttling of intermediates across the membranes not only once but twice, is rather intriguing. Because the arabinose contents in the *mur4* knock-out mutants and the *rgp1/2* knock-down mutants were severely reduced [51], [53], the possibility of the presence of other epimerases in the cytosol or mutases in the Golgi lumen is unlikely. Therefore, together with the previous reports [52], [53], our results provide strong evidence of the existence of novel mechanisms of glycan biosynthesis involving intermediate shunts across the membrane (Figure 9).

The TMD topology reporters successfully detected the membrane topologies of all eleven proteins tested. This was initially unexpected because BiFC is also used to detect specific protein-protein interactions [51], [53]. The apparent promiscuous fluorescence complementation by the TMD reporters could be attributed to the high level of expression under the experimental conditions tested. This is likely to result in ubiquitous distribution and high concentrations of the reporters across these subcellular compartments, thereby facilitating non-specific interaction between the reporters and the tested proteins. Additionally, the N- and C-terminal portions of TMD are predicted to be disordered by the three web-based programs, DisEMBL [56], DISOPRED [57], and GlobPlots [58]. Intrinsically disordered regions are thought to be structurally extended and flexible, which enhances the initial, relatively non-specific, associations that occur in protein-protein interactions [59]. It is plausible that the promiscuous fluorescence complementation between the TMD reporters and all the proteins tested is partly attributed to the disordered termini of TMD. Disordered regions may enhance the irreversible interaction between the split halves of YFP molecules [60], rendering the TMD-based reporters very robust.

It is noteworthy that the N-terminal fusions of tested proteins (XXT1, XXT2, XXT5 and MUR3) caused localization not only to the Golgi apparatus but also to ER, even though the C-terminal fusions of the same tested proteins localized predominantly in the Golgi apparatus (Figure 7). It is plausible that the partial ER localization is an artifact due to a combination of the presence of the tag in the N-termini and the effect of over-expression under the present conditions. In general, for protein topology analysis that relies on the tagging of protein termini, N-terminal tagging should be avoided. The N-termini of some proteins contain signal peptides that are cleaved after translation. In this case, the detected topology bears no information about the topology of the mature proteins. In addition, we have observed that YFP tagged to the N-termini of proteins and localized to the cytosol was partially degraded (Figure 3, Figure 6) or released from the fused protein (Figure 7, see Yn-MUR3 co-expressed with Yc). Lastly, the presence of a structured peptide (e.g. the Yn or Yc tag) preceding the first transmembrane domain may alter the overall topology of membrane proteins such as type I membrane proteins and multimembrane spanning proteins that insert N-terminal ends into the lumen of the secretory pathway. Most membrane proteins are co-translationally translocated across the membrane by the translocation channel, the Sec61 complex [61], [62]. The first transmembrane domain, as it emerges from the ribosome, intercalates into the lateral gate of the channel complex. The N-terminus flips across the channel and subsequently exits the translocon laterally into the lumen if the hydrophobic region is long and the preceding segment does not contain positive charges or stable folding [62]. Otherwise the N-terminus is retained in the cytosol and the proceeding polypeptide is elongated and the C-terminus is translocated across the channel. To date, it is not clear to what extent the tagging of N-termini of membrane proteins with structured polypeptide tags impact the membrane topology. A systematic and thorough analysis addressing the impact of sizes and folding of tags on the N-terminal translocation across the membrane is needed.

This study developed and validated GO-PROMTO as a highly robust and easy method for determining topology of membrane proteins in the Golgi apparatus and ER in complex living tissues of higher plants. By using GO-PROMTO the membrane topology of xyloglucan and arabinan biosyntheses were examined. Even though GO-PROMTO was only tested in plant tissue, it can potentially be used in other organisms. Large-scale analysis of membrane protein topology has been performed previously in *Escherichia coli* and *Saccharomyces cerevisiae* [13], [14]. GO-PROMTO may be readily and universally adapted to global topology analyses in higher eukaryotes, either in cell cultures or in intact tissues. The binary signal output makes the detection and interpretation of the results straightforward and suitable for high-throughput applications.

## Materials and Methods

### Vector constructions and transformation of *Agrobacterium tumefaciens*

In-frame fusions of the *Arabidopsis thaliana* cDNA CSLD2 (At5g16910), ARAD1 (At2g35100), RGXT2 (At4g01750), UXS2 (At3g62830), UGE4 (At1g64440), MUR3 (At2g20370) and MUR4 (At1g30620) to Yn or Yc tags and that of XXT5 (At1g74380) to Yc were generated in pCAMBIA330035su using the USER cloning technique [63]. The design of the linker sequence was identical in all constructs as described previously [29]. In-frame fusions of the Arabidopsis cDNA XXT1 (At3g62720), XXT2 (At4g02500) and CSLC4 (At3g28180) to Yn, and Yc as well as that of XXT5 to Yn were carried out by Gateway cloning strategy according to the manufacturer's protocol (Invitrogen). The following destination vectors were used: the pEarlygate 104 vector for full length YFP fusions [64]; and Vyne or Vyce Gateway vectors for Yn and Yc, respectively [65]. *Agrobacterium tumefaciens* pGV3850 C58C01 [65] was transformed with plasmids bearing the in-frame fusions by electroporation and the transformants were selected in the presence of appropriate antibiotics. The transformants were stored at −80°C until used.

### Transient expression in tobacco

Strains were grown in LB or YEP media with appropriate antibiotics overnight at 28°C with agitation at 220 rpm. After centrifugation, cell pellets were resuspended in 1 ml infiltration buffer containing 10 mM MES, pH 5.6, 100 µM acetosyringone and 10 mM MgCl_2_ at the final optical density at 600 nm of 0.05. The viral suppressor of gene silencing, p19, [66] was co-infiltrated at an final optical density at 600 nm of 0.1 in combinations containing XXT1, XXT2, XXT5 or MUR3. Transient expression of the fusion proteins were carried out in 4-week-old *Nicotiana benthamiana* plants that have been grown in greenhouses at 24°C/17°C day/night temperatures, 16 hour photoperiod. Each strain combination was infiltrated into a separate leaf, in two independent plants. Infiltrations were done by injection on the bottom face of the leaf using a syringe without a needle, with a fingertip providing counter pressure. After infiltration, the plants were placed in the greenhouse for 3–4 days until observations by microscopy. At least two individual experiments were performed for each combination.

### Confocal laser scanning microscopy

CLSM used in this study were Leica SP5 II and SP5-X AOBS CLSM with appropriate Leica Application Suite Advanced Fluorescence software as previously described [29]. Samples were excited with a 40% 514 nm Argon laser line. The emission of YFP was detected between 525 and 599 nm. Overall gain settings were in the range of 600 to 850 volts, and the gain setting was kept constant for each glycan biosynthetic fusion protein. The samples were observed with a Leica 40×/0.8 Numerical Aperture (NA) dipping lens with milliQ water as immersion media. Image processing, where it was necessary, was performed by using Adobe Photoshop CS3.0 Extended v10.0.

### Protease protection assay and western blot of TMD constructs

Two to four infiltrated leaves at 2–4 days post infiltration (DPI) were harvested. Microsomes were prepared as follows. Leaves were frozen and ground in liquid nitrogen. The ground tissue was macerated in an extraction buffer, consisting of 100 mM HEPES-KOH pH 7.25, 300 mM sucrose, 5 mM EGTA, 5 mM MgCl_2_ and 1 Complete Protease Inhibitor tablet (Roche) per 50 ml extraction buffer. The homogenates were subjected to centrifugation for 3000× g in a F15 rotor (Piramoon Technologies) for 15 min at 4°C, followed by ultracentrifugation of the supernatants at 113,000× g in a SW40 Ti rotor (Beckman Coulter) for 1 h at 4°C. The pellets were resuspended in the extraction buffer by light brushing with a small paint brush. The protein content of each sample was determined by the standard Bradford assay [67].

The protease protection assay was carried out as follows. Ten micrograms of protein from each sample was mixed with either Triton X-100, proteinase K (freshly made from a lyophilized stock, Sigma P6556) or both in the final volume of 15 µl. Final concentrations was 0.1% (v/v) and 0.1 µg µl^−1^, respectively. The mix was incubated at 30°C for 30 min. The reaction was stopped by the addition of phenylmethanesulfonylfluoride at the final concentration of 6 mM. The samples were mixed with 5-fold strength sample buffer (0.125 M Tris-HCl, pH 6.8, 20% (v/v) glycerol, 4% (v/v) SDS, 1% (v/v) bromophenol blue) containing 200 mM dithiothreitol. The mixed samples were treated for 10 min at 55°C and proteins were separated in Criterion XT pre-cast 12% (v/v) bis-Tris gels with MOPS buffer (Bio-Rad). Immunoblots were carried out with Protran nitrocellulose transfer membrane (Whatman) in a full wet Criterion blotting system (Bio-Rad). The primary antibody, rabbit anti-GFP antibody (A11122, Invitrogen) was diluted by 5000 folds, and the secondary antibody, anti-rabbit horse radish peroxidase conjugated antibody (Dako PO217, DAKO Denmark, DK), was diluted by 1000 folds. The horse radish peroxidase signal was detected with a chemiluminescent detection system (Super-Signal; Pierce) according to the instructions of the manufacturer using an Autochemi UVP system (AH Diagnostics) with LabWorks version 4.5 software.
